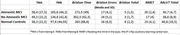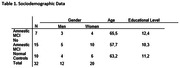# Utility of Reading The Mind in the Eyes test and Brixton Spatial Anticipation Test in the assessment of mild cognitive impairment in underrepresented populations

**DOI:** 10.1002/alz.095485

**Published:** 2025-01-09

**Authors:** Francesca Mariani

**Affiliations:** ^1^ Hospital de Clínicas, Montevideo, Montevideo Uruguay

## Abstract

**Background:**

The choice of tests and batteries for cognitive evaluation in neuropsychology is biased by a central aspect of each patient, their educational level. This can be very different between cultures, and is particularly low in some regions of Latin America. This population characteristic generates limitations in the tests that we can perform in some patients with a low educational level. For example, the use of the TMB, which requires proper use of the alphabet. We evaluate the utility and receptivity of The Brixton Spatial Anticipation Test and the Reading in the Mind Eyes test in patients with mild cognitive impairment of underrepresented populations.

**Method:**

We evaluated the performance on the Brixton and RMET in a sample of 22 MCI patients (7 amnesic and 15 no amnestic), and 10 healthy controls and compare it with other typical cognitive measures.

**Result:**

Demographic characteristics of study subjects are shown in Table 1. The MCI amnestic group perform better than the MCI no amnestic group in RMET and Brixton test. With a lower execution time in the last one. Also, we found for the MCI no amnestic group a positive and moderate correlation between Brixton execution time and TMB. For de MCI amnestic group we found a positive and moderate correlation between the number of errors in Brixton and de RMET performance. Finally the MCI amnestic group shows a negative and moderate correlation between RMET and total of RALVT.

**Conclusion:**

Our results suggests that the Brixton Spatial Anticipation Test could be a very good option for executive functioning measures, and sensitive enough to be used in cases that gold standard test couldn´t be applied. Also we can suggest that affective theory of mind test like RMET could be a useful assessment tool for MCI patients. It is necessary to perform longitudinal studies in order to being able to conclude about the performance in this test in the evolution of these type of patients and it role in differential diagnosis.